# Mesenchymal Stromal Cells Are More Immunosuppressive *In Vitro* If They Are Derived from Endometriotic Lesions than from Eutopic Endometrium

**DOI:** 10.1155/2017/3215962

**Published:** 2017-11-05

**Authors:** Fawaz Abomaray, Sebastian Gidlöf, Cecilia Götherström

**Affiliations:** ^1^Division of Obstetrics and Gynecology, Department of Clinical Science, Intervention and Technology, Karolinska Institutet, Stockholm, Sweden; ^2^Centre for Hematology and Regenerative Medicine, Karolinska Institutet, Stockholm, Sweden; ^3^Department of Obstetrics and Gynecology, Karolinska University Hospital, Stockholm, Sweden; ^4^Department of Women's and Children's Health, Karolinska Institutet, Stockholm, Sweden

## Abstract

Endometriosis is an inflammatory disease with predominance of immunosuppressive M2 macrophages in the pelvic cavity that could be involved in the pathology through support and immune escape of ectopic lesions. Mesenchymal stromal cells (MSC) are found in ectopic lesions, and MSC from nonendometriosis sources are known to induce M2 macrophages. Therefore, MSC were hypothesized to play a role in the pathology of endometriosis. The aim was to characterize the functional phenotype of MSC in ectopic and eutopic endometrium from women with endometriosis. Stromal cells from endometriotic ovarian cysts (ESC_cyst_) and endometrium (ESC_endo_) were examined if they exhibited a MSC phenotype. Then, ESC were phenotypically examined for protein and gene expression of immunosuppressive and immunostimulatory molecules. Finally, ESC were functionally examined for their effects on monocyte differentiation into macrophages. ESC_cyst_ and ESC_endo_ expressed MSC markers, formed colonies, and differentiated into osteoblasts and adipocytes. Phenotypically, ESC_cyst_ were more immunosuppressive, with significantly higher expression of immunosuppressive molecules. Functionally, ESC_cyst_ induced more spindle-shaped macrophages, with significantly higher expression of CD14 and CD163, both features of M2 macrophages. The results suggest that ESC_cyst_ may be more immunosuppressive than ESC_endo_ and may promote immunosuppressive M2 macrophages that may support growth and reduce immunosurveillance of ectopic lesions.

## 1. Introduction

Endometriosis is an inflammatory disease where the endometrium grows in ectopic sites, most commonly in the pelvic cavity [1]. The major symptoms of endometriosis are chronic pelvic pain and infertility [2]. Although medical and surgical treatments are available, they are not sufficient as recurrence of ectopic lesions and symptoms is common [3]. The mechanism behind disease development, progression, and recurrence is not fully known. Therefore, there is a need for improved understanding of the pathology of endometriosis.

Sampson's theory of retrograde menstruation is the most widely accepted theory for the pathophysiology of endometriosis [2] and states that reflux of menstrual debris during menstruation implants in the pelvic cavity and causes endometriosis [4]. Almost all women have retrograde menstruation, but only approximately 10% develop this disease [5], indicating that endometriosis has a multifactorial pathogenesis with other factors involved, such as reduced immunosurveillance in the pelvic cavity of women with endometriosis and involvement of stem/stromal cells [2, 6]. The latter factor refers to the stem cell theory, which postulates that a putative stem/stromal cell population such as mesenchymal stromal cells (MSC) is refluxed back into the pelvic cavity via retrograde menstruation and then gives rise to ectopic lesions [6].

MSC are multipotent cells that are known to exist in both ectopic lesions and in the endometrium [6]. They have attracted much attention in the last decade mainly for their capacity to modulate immune responses [7]. Accordingly, they have been suggested as a potential treatment for inflammatory diseases such as graft versus host disease, multiple sclerosis and type 1 diabetes, among others [8]. Moreover, it would be expected that MSC are found in ectopic lesions through retrograde menstruation.

High levels of proinflammatory cytokines such as interferon-*γ* (IFN-*γ*) and tumour necrosis factor-*α* (TNF-*α*) are found in the pelvic cavity of women with endometriosis [9]. Moreover, ectopic lesions behave like eutopic endometrium in the menstrual cycle stimulated to grow and then shed with fluctuations in the levels of steroid hormones furthermore promoting inflammation [2, 10]. MSC can sense and respond to inflammation in their microenvironment [11–13]. In fact, it has been suggested that high levels of inflammation polarize them into an immunosuppressive phenotype expressing high levels of immunosuppressive molecules such as indoleamine 2,3-dioxygenase 1 (IDO1), cyclooxygenase 2 (COX2), and heme oxygenase 1 (HO-1) leading to immunosuppression via promotion of immunosuppressive M2 macrophages [13]. In contrast, low levels of inflammation have been suggested to polarize them into an immunostimulatory phenotype expressing high levels of proinflammatory cytokines and chemokines such as interleukin 8 and C-X-C motif chemokine 12 (CXCL12) which cause immunostimulation via immune cell recruitment and promotion of immunostimulatory M1 macrophages [13]. Studies show that M2 macrophages, immature dendritic cells, and T helper 2 (TH2) responses predominate in ectopic lesions [14–16]. In addition, cytotoxic functions of natural killer and CD8 T cell activities are inhibited and regulatory T cell activities are induced [17–19]. Moreover, MSC from nonendometriosis sources are known to promote these processes [20–25] which suggest that MSC in ectopic lesions may be immunosuppressive.

Accordingly, the aim of this study was to characterize the functional phenotype of MSC in ectopic and eutopic endometrium isolated from women with endometriosis. We hypothesized that immunosuppressive MSC may predominate in ectopic lesions contributing to their reduced immunosurveillance and growth. Stromal cells from ectopic (ESC_cyst_) and eutopic (ESC_endo_) endometrium were examined if they exhibited MSC characteristics. Their phenotypes were examined by determining their expression of several immunosuppressive and immunostimulatory markers, and finally, their functional effects on differentiation of monocytes into macrophages were examined. Both sources of stromal cells were found to be MSC, but ESC_cyst_ displayed more phenotypically and functionally immunosuppressive characteristics. The data suggest that ESC_cyst_ may promote immunosuppressive M2 macrophages that may support and reduce immunosurveilance of ectopic lesions allowing their growth.

## 2. Materials and Methods

### 2.1. Human Tissue Samples

Two types of tissues were collected: (i) endometriotic ovarian cysts (ectopic endometrium) and (ii) endometrium from women with endometriosis (eutopic endometrium). The endometriotic ovarian cysts and endometrium were collected from women aged from 31 to 42 years (mean ± SD, 36.3 ± 5.8 years, *n* = 4) undergoing laparoscopic surgery for confirmation or treatment of endometriosis. All women were histologically confirmed to have endometriosis by a pathologist. Only one woman underwent hormonal treatment. Moreover, two of the biopsies were from the proliferative phase: one was unknown and one had amenorrhea. Informed oral and written consent was obtained from each participant, and ethical approval was obtained from The Regional Ethical Review Board in Stockholm (2013/1094-31/2).

### 2.2. Isolation of Stromal Cells from Eutopic and Ectopic Endometrium

Human endometrial and endometriotic ovarian cyst tissues were digested to a single cell suspension using 1 mg/mL collagenase type I (Sigma, Missouri, United States) diluted in Hank's balanced salt solution (Life Technologies, Paisley, UK) (90 minutes for endometriotic tissue and 30 minutes for endometrial tissue) at 37°C with shaking every 10 minutes. The tissue digests were filtered twice through 100 *μ*m cell strainers (Corning, New York, United States), and eventually the stromal cells were filtered through a 40 *μ*m cell strainer (Corning), with undigested tissue and epithelial cells being removed at each of the steps. The cell suspension was washed twice with phosphate-buffered saline (PBS) (Life Technologies), by centrifugation at 500 ×g for 10 minutes. Finally, the cell pellet was resuspended in complete growth medium containing Dulbecco modified essential medium low glucose (DMEM-LG) (Life Technologies) + 10% MSC certified fetal calf serum (FCS) (Life Technologies) + 1% antibiotic and antimycotic (Life Technologies). Viable cells were counted in 1% Eosin (Merck KGaA, Darmstadt, Germany) and cultured at 4000 cells/cm^2^ in tissue culture flasks at 37°C with 5% CO_2_. After two days, the growth medium was changed and thereafter every three to four days. When the cells reached 70–90% confluency, they were trypsinised using 0.05% trypsin/EDTA (Life Technologies) and cultured as described above. At passage 2, the stromal cells were cryopreserved in 10% dimethyl sulfoxide (DMSO) (Sigma) in complete growth medium. To ensure that we were working with a pure population of cells, ESC_endo_ and ESC_cyst_ were used at passages three to six, as earlier passages may be contaminated with other cell types.

### 2.3. MSC Characterization Using Flow Cytometry

ESC_endo_ and ESC_cyst_ were stained with antibodies against CD73 PE (Becton-Dickinson, New Jersey, United States), CD90 PerCP Cy5 (BioLegend, California, United States), CD105 FITC (Ancell, Minnesota, United States), HLA class I PE (Agilent, Stockholm, Sweden), HLA class II FITC (Agilent), CD14 FITC (Becton-Dickinson), CD45 APC (BioLegend), and CD31 APC (BioLegend) for 20 minutes at room temperature (RT). Then, they were washed twice with PBS by centrifugation at 500 ×g for 10 minutes. Finally, the cells were resuspended in PBS with 0.1% bovine serum albumin (Sigma) and analyzed with BD FACSCalibur (Becton-Dickinson). Unstained controls were used to set gates and voltages. The data was analyzed using the software Flow-Jo (Tree Star Inc., Ashland, United States). When the percentage of cells expressing a particular marker was ≥95% or ≤5%, then they were termed positive or negative for that marker, respectively.

### 2.4. Colony-Forming Units Fibroblasts (CFU-F)

Colony-forming efficiency of ESC_endo_ and ESC_cyst_ was assessed using CFU-F in which cells were seeded in six-well plates at optimized densities of 200 cells/well in complete DMEM-LG growth medium. The growth medium was changed every 4 days. Following 21-day culture in 37°C with 5% CO_2_, the growth medium was removed and the cells were washed twice with PBS. The cells were then fixed and permeabilized with 100% cold methanol for 5 minutes at RT. After washing the cells twice with PBS, they were stained with 1% Eosin in PBS for 20 minutes at RT, then rinsed twice with milliQ water, and visualized at 4x magnification under an Olympus CKX41 inverted microscope (Olympus, Tokyo, Japan). Colonies of cell aggregates of ≥50 cells were scored in the whole wells.

### 2.5. Osteogenic Differentiation

For differentiation into osteoblasts, stromal cells at a density of 5 × 10^3^ cells/cm^2^ were seeded in 12-well plates and cultured at 37°C with 5% CO_2_ until they reached 50–70% confluency. After removing the growth medium, cells were washed twice with PBS and then induced to differentiate into osteoblasts using osteoblast differentiation medium containing complete DMEM-LG, 10 nM dexamethasone (Sigma), 10 mM *β*-glycerophosphate (Sigma), and 0.05 mM ascorbic acid (Sigma). After 14–21 days culture, the growth medium was removed, cells were then washed twice with PBS, and fixed with 4% paraformaldehyde (PFA) (Sigma) for 30 minutes at RT. Then cells were washed twice with PBS and stained with 2% of Alizarin red S (Sigma) at pH 4.1–4.3 for 10 minutes at RT with gentle rotation. After washing the cells 5 times with milliQ water, then 15 min with PBS, they were visualized under an Olympus CKX41 inverted microscope, and images were then captured. For quantitation of the calcium salts stained by Alizarin red S, the Alizarin red S dye was eluted with 10% cetylpyridinium chloride (CPC) (Sigma) in milliQ water for 15 minutes at RT with gentle rotation. The absorbance was measured using the Infinite F200 PRO Tecan spectrophotometer (Tecan, Mannedorf, Switzerland) at 570 nm. 10% CPC was used as a blank.

### 2.6. Adipogenic Differentiation

For adipocyte differentiation, stromal cells at a density of 2 × 10^4^ cells/cm^2^ were seeded in 12-well plates and cultured at 37°C with 5% CO_2_ until they reached 100% confluency. After removing the growth medium, cells were washed twice with PBS and then were induced to differentiate into adipocytes using an induction medium of complete growth medium containing DMEM high glucose (DMEM-HG) (Life Technologies), 10% FCS, 1% A/A, 1 *μ*M dexamethasone, 0.2 mM indomethacin (Sigma), 0.5 mM 3-isobutyl-1-methylxanthine (Sigma), and 0.01 mg/mL insulin (Life Technologies). After 3 days, support medium containing DMEM-HG, 10% FCS MSC, 1% A/A, and 0.01 mg/mL insulin was added for another 1–3 days. This induction and support medium cycle was repeated 3 times, and then the cells were cultured for 7 days in support medium. Following differentiation, cells were washed twice with PBS and fixed with 4% PFA for 60 minutes at RT. Then, the cells were washed twice with milliQ water, and 60% isopropanol (Sigma) was added for 5 minutes at RT. Afterwards, the cells were stained with Oil red O (Sigma) for 10 minutes at RT. Finally, the cells were washed 4 times with milliQ water, visualized under an Olympus CKX41 inverted microscope, and images were captured. For quantitation of the lipid vacuoles staining, the Oil red O dye was eluted with 100% isopropanol. The absorbance was measured using the Infinite F200 PRO Tecan spectrophotometer at 492 nm. 100% isopropanol was used as a blank.

### 2.7. Phenotypic Characterization of ESC by Flow Cytometry

The protein expression of IDO1, COX2, HO-1, and CXCL12 was determined by flow cytometry. ESC_endo_ and ESC_cyst_ were cultured at 1 × 10^4^ cells/cm^2^ until ~90% confluency, and then they were harvested using 0.05% trypsin/EDTA. For CXCL12, 5 hours before the end of culture and harvesting, cells were treated with golgi plug (Becton-Dickinson). Then the cells were fixed with 4% PFA for 10 minutes at RT, washed twice with PBS, and permeabilized using 0.1% saponin (USB, Buckinghamshire, UK) for 15 minutes at RT. Cells were then washed twice with PBS and stained with IDO PE (Bio-Techne, Minnesota, United States), COX2 Alexa fluor 488 (Cell Signaling Technologies, Massachusetts, United States), HO-1 APC (US Biological, Massachusetts, United States), and CXCL12 Alexa Fluor 488 (Novus Biologicals, Colorado, United States) in 0.1% saponin for 20 minutes in the dark at RT. Afterwards, the cells were washed twice with 0.1% saponin, resuspended in PBS with 0.1% BSA, and then run on the BD LSR Fortessa (Becton-Dickinson). Unstained cells were used to set gates and voltages. The data was analyzed using the software Flow-Jo.

### 2.8. Phenotypic Characterization of ESC by Quantitative Polymerase Chain Reaction (qPCR)

To determine the gene expression of the IDO1, COX2, HO-1, and CXCL12 (genes listed in Table 1), qPCR was performed. Beta-actin (*β*-actin) was used as a housekeeping gene control. Forward and reverse primers were designed as instructed by Eurofins genomics and used according to the manufacturer's instructions. Cells previously stored in RNA*later* (ThermoFisher Scientific, Massachusetts, United States) at −80°C were thawed, diluted with an equal volume of PBS, and centrifuged at 500 ×g for 10 minutes, and the supernatant was then carefully removed. The resulting cell pellets were used to isolate total RNA using the RNeasy mini kit (Qiagen, Hilden, Germany) according to the manufacturer's instructions. Then, the RNA purity and concentration were measured using the nanodrop 2000c spectrophotometer (ThermoFisher Scientific). 100 ng/*μ*L RNA was used to synthesize cDNA using the High capacity cDNA reverse transcription kit (Applied Biosystems, Vilnius, Lithuania) according to the manufacturer's instructions. Finally, gene expression was quantified using the Fast SYBR green master mix (Applied Biosystems) according to the manufacturer's instructions, and the reactions were carried out in triplicate on the qPCR CFX384 real-time system C1000 touch thermal cycler (Bio-Rad, Stockholm, Sweden). Then, the data was analyzed using the software CFX manager (Bio-Rad). The relative expression level of the housekeeping gene *β*-actin was used to normalize target gene expression, and gene expression between ESC_endo_ and ESC_cyst_ was analyzed using the comparative Ct method (∆∆Ct method), using ESC_endo_ as the calibrator.

### 2.9. Isolation of Human Monocytes

Peripheral blood mononuclear cells (PBMCs) were isolated from buffy coats from healthy donors using SepMate tubes (StemCell Technologies, Cambridge, United Kingdom) and Lymphoprep gradient separation according to the manufacturer's instructions (Axis-Shield). Then, monocytes were isolated (*n* = 3) using the Monocyte isolation kit II (Miltenyi Biotech, Lund, Sweden) and a magnetic cell separation system (Miltenyi Biotech) as described previously [26]. The PBMCs were magnetically labelled with a cocktail of biotin-conjugated antibodies against CD3, CD7, CD16, CD19, CD56, CD123, and Glyocophorin A and antibiotin microbeads. Then, untouched monocytes were isolated by passing PBMCs through a column placed in a magnetic cell separator according to the manufacturer's instructions and depleting the magnetically labelled cells. The purity of the isolated monocytes was assessed by flow cytometry using an anti-CD14 monoclonal antibody (Becton-Dickinson); samples with purity ≥ 95% were used for experiments.

### 2.10. Functional Characterization of ESC

When the confluency for ESC_endo_ and ESC_cyst_ was ~70%, the growth medium was removed, the cells were washed twice with PBS, and fresh growth medium was added. After three days, the conditioned medium (CM) was collected, centrifuged at 500 ×g for 10 minutes to remove cellular debris, aliquoted, and frozen at −80°C. The CM was used for subsequent experiments (60% CM and 40% growth medium as described below). The untouched human monocytes were cultured in Roswell Park Memorial Institute (RPMI) (Life Technologies) 1640 growth medium, 10% FCS, 1% L-glutamine, 1% penicillin and streptomycin, and CM from ESC_endo_ or ESC_cyst_ from day 0 for 7 days. Then, images were acquired with the Olympus CKX41 inverted microscope at 20x magnification, and the cells were harvested for flow cytometry analysis. The monocytes were stained with CD14 FITC (Becton-Dickinson), CD163 PE (Becton-Dickinson), and CD206 FITC (Becton-Dickinson) for 20 minutes in the dark at RT. Then, the cells were washed twice with PBS by centrifugation at 500 ×g for 10 minutes, finally resuspended in PBS with 0.1% BSA, and analyzed with BD FACSCalibur. Unstained cells were used to set gates and voltages. The data was analyzed using the software Flow-Jo.

### 2.11. Statistical Analysis

All statistical analyses were performed using GraphPad prism 6. When data was normally distributed, the means were analyzed with Student *t*-test, and when it was not normally distributed, the medians were analyzed with the Mann–Whitney test. All values are shown as the mean ± standard deviations (SD). For the study, *n* refers to the number of biological replicates. Results were considered to be statistically significant if *P* < 0.05.

## 3. Results

### 3.1. MSC Exist in both Ectopic and Eutopic Endometrial Tissue

**Figure 1:**
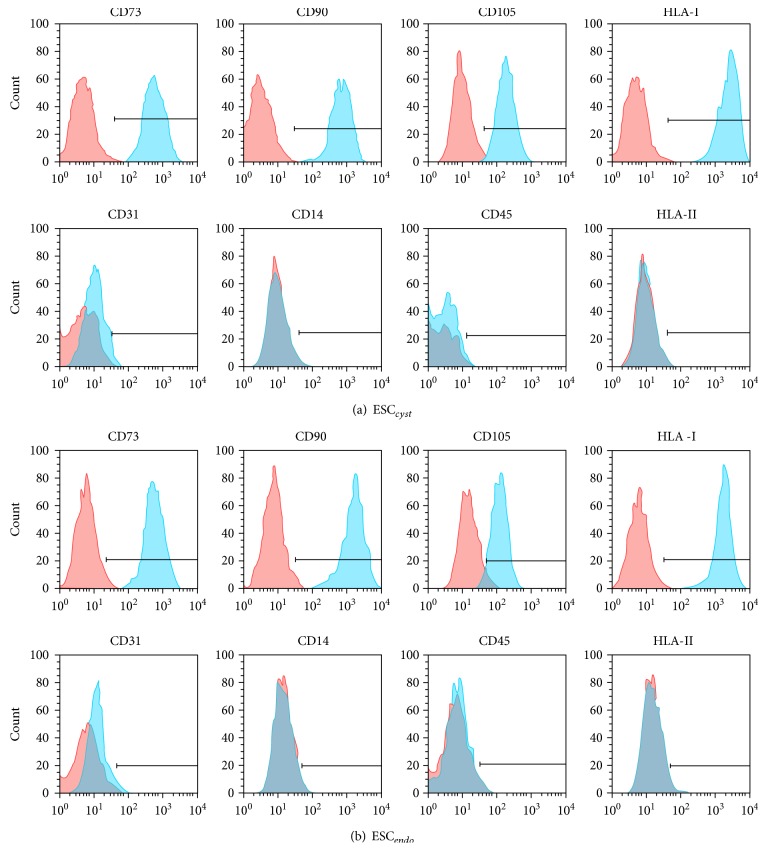
Representative flow cytometry histograms for ESC_endo_ and ESC_cyst_, which were positive (≥95%) for the MSC markers CD73, CD90, CD105, and HLA class I and negative (≤5%) for the non-MSC markers CD14, CD45, CD31, and HLA class II. Unstained control cells are in red, and stained cells are in blue. The horizontal lines on the histograms show the percentage of expression of the markers compared to the unstained control cells. Five to seven independent experiments (*n* = 3 biological replicates) were carried out.

**Figure 2:**
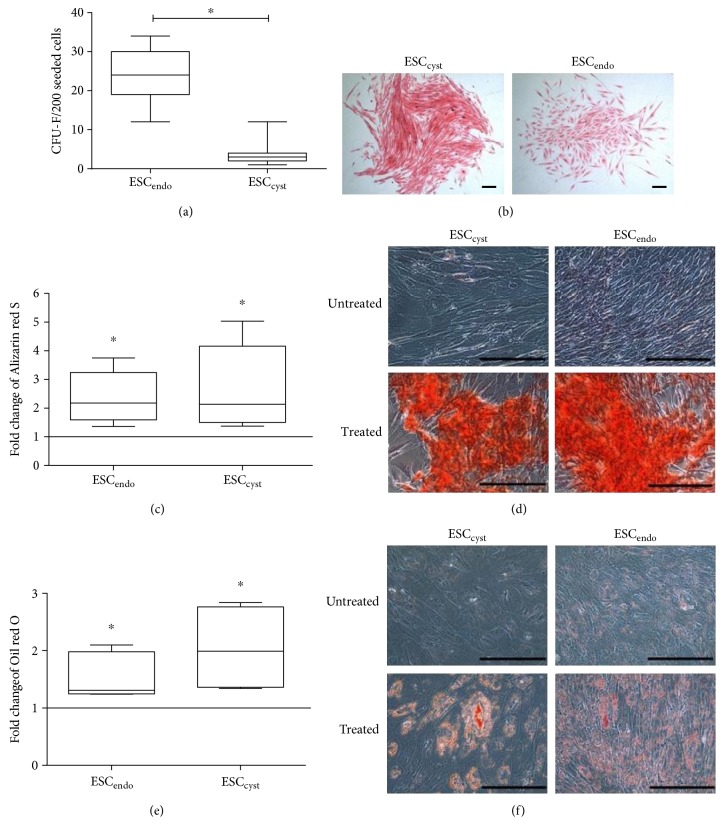
Colony-forming unit fibroblasts for ESC_endo_ and ESC_cyst_ after they were cultured at clonal density for 21 days. Also, osteoblast differentiation for ESC_endo_ and ESC_cyst_ following 14–21 days culture in osteoblast differentiation growth medium and adipocyte differentiation for ESC_endo_ and ESC_cyst_ following 28-day culture in adipocyte differentiation growth medium are shown. The colony-forming efficiency (a) shows that ESC_endo_ formed significantly more colonies than the ESC_cyst_ (^∗^*P* < 0.05). Representative phase contrast images (b) at 4x magnification following Eosin staining of ESC_endo_ and ESC_cyst_. Colonies with ≥50 cells were counted. Quantitation of the Alizarin red S dye (c) as a mean fold change relative to the untreated controls; both types of stromal cells significantly induced osteoblast differentiation (^∗^*P* < 0.05). Representative phase contrast images (d) at 20x magnification following Alizarin red S staining of the calcium salts. Quantitation of the Oil red O dye (e) as a mean fold change relative to the untreated controls; both types of stromal cells significantly induced adipocyte differentiation (^∗^*P* < 0.05). Representative phase contrast images (f) at 20x magnification following Oil red O staining of the lipid vacuoles. Nine independent experiments (*n* = 3 biological replicates) were carried out in triplicates for (a, b). Three to four independent experiments (*n* = 3 biological replicates) were carried out in duplicates for (c, d, e, f). Mean ± SD. Scale bars represent 50 *μ*m at 4x magnification for (a,b) and 50 *μ*m at 20x magnification for (c, d, e, f).

To verify that ESC_endo_ and ESC_cyst_ had the phenotype of MSC, we examined the cells by flow cytometry, CFU-F, and differentiation assays into osteoblasts and adipocytes. ESC_endo_ and ESC_cyst_ expressed the MSC markers CD73, CD90, CD105, and HLA class I, but did not express the non-MSC markers CD14, CD45, CD31, and HLA class II (Figures 1(a) and 1(b)). ESC_endo_ and ESC_cyst_ were both able to form colonies, albeit at a significantly lower (*P* < 0.05) efficiency for ESC_cyst_ (Figures 2(a) and 2(b)). Furthermore, stromal cells from both sources were able to differentiate into osteoblasts and adipocytes significantly (*P* < 0.05) as compared to untreated controls (*P* < 0.05) (Figures 2(c), 2(d), 2(e), and 2(f)). Taken together, the results indicate that both ESC_endo_ and ESC_cyst_ are MSC.

### 3.2. ESC_cyst_ Have a More Immunosuppressive Phenotype than ESC_endo_

**Figure 3:**
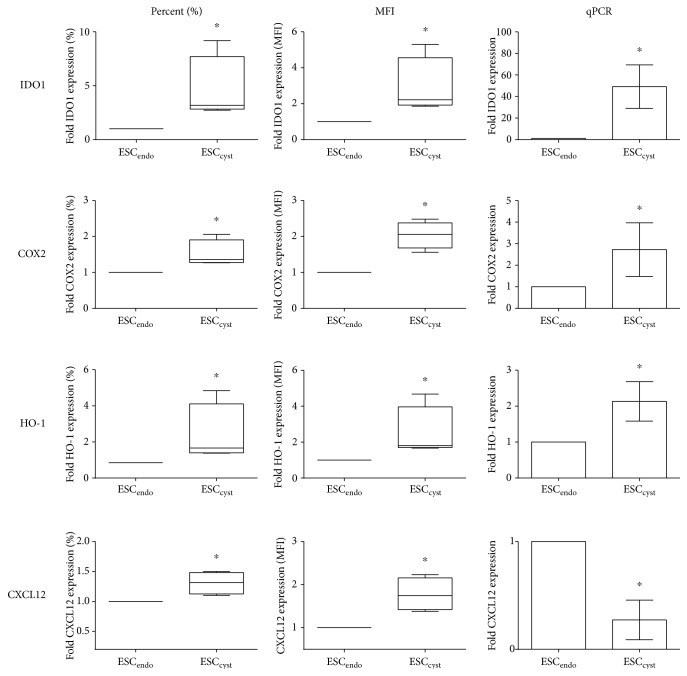
Protein and gene expression of the immunosuppressive enzymes IDO1, COX2, and HO-1 and the proinflammatory chemokine CXCL12 in ESC_endo_ and ESC_cyst_ by flow cytometry and qPCR, respectively. The percentage of cells expressing IDO1 is higher for ESC_cyst_ (^∗^*P* < 0.05). ESC_cyst_ express higher levels of IDO1 by MFI (^∗^*P* < 0.05). ESC_cyst_ have higher gene expression of IDO1 (^∗^*P* < 0.05). The percentage of cells expressing COX2 is higher for ESC_cyst_ (^∗^*P* < 0.05). ESC_cyst_ express higher levels of COX2 by MFI (^∗^*P* < 0.05). ESC_cyst_ have higher gene expression of COX2 (^∗^*P* < 0.05). The percentage of cells expressing HO-1 is higher for ESC_cyst_ (^∗^*P* < 0.05). ESC_cyst_ express higher levels of HO-1 by MFI (^∗^*P* < 0.05). ESC_cyst_ have higher gene expression of HO-1 (^∗^*P* < 0.05). The percentage of cells expressing CXCL12 is higher for ESC_cyst_ (^∗^*P* < 0.05). ESC_cyst_ express higher levels of CXCL12 by MFI (^∗^*P* < 0.05). ESC_cyst_ have lower gene expression of CXCL12 (^∗^*P* < 0.05). Four independent experiments (*n* = 4 biological replicates) were carried out in duplicates. Mean ± SD.

To characterize ESC_endo_ and ESC_cyst_ phenotypically, the gene and protein expressions of the immunosuppressive enzymes IDO1, COX2, and HO-1 and the proinflammatory chemokine CXCL12 were examined by both flow cytometry and qPCR, respectively. By flow cytometry, ESC_cyst_ expressed higher levels of IDO1, COX2, and HO-1 compared to ESC_endo_. The percentage of ESC expressing these immunosuppressive enzymes and the level of expression or the median fluorescence intensity (MFI) in the positive cells was significantly higher (*P* < 0.05) for ESC_cyst_ compared to ESC_endo_ (Figure 3). Moreover, gene expression as analyzed by qPCR showed that IDO1, COX2, and HO-1 was expressed significantly higher (*P* < 0.05) in ESC_cyst_ compared to ESC_endo_ (Figure 3).

Flow cytometry analysis showed that ESC_cyst_ expressed higher levels of CXCL12 than ESC_endo_; the percentage of stromal cells expressing CXCL12 and the MFI was significantly higher (*P* < 0.05) for ESC_cyst_ as compared to ESC_endo_ (Figure 3). However, gene expression of CXCL12 was significantly lower (*P* < 0.05) for ESC_cyst_ compared to ESC_endo_ by qPCR (Figure 3).

### 3.3. ESC_cyst_ Have a More Immunosuppressive Function than ESC_endo_

**Figure 4:**
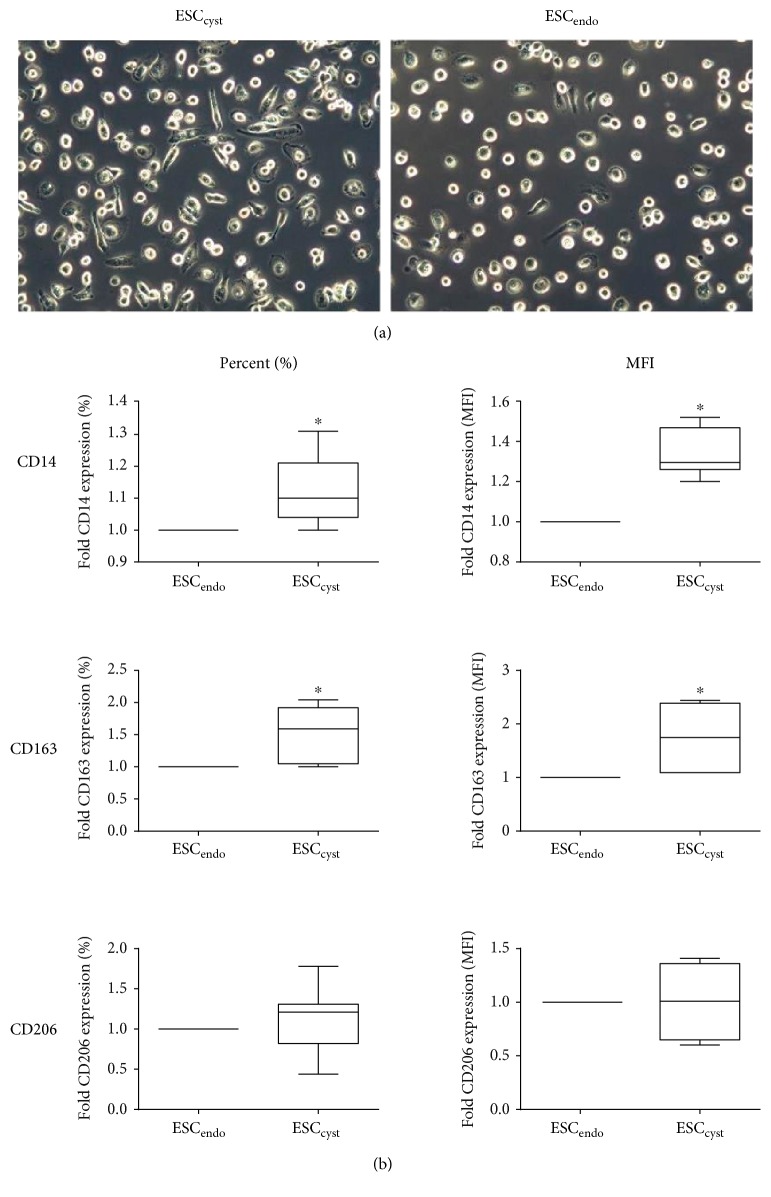
The effects of ESC_endo_ and ESC_cyst_ conditioned medium (CM) on freshly isolated untouched human monocytes. Representative phase contrast images (a) of the CM-treated monocytes after 7 days of culture at 20x magnification. The percentage of macrophages expressing CD14 and CD163 and the level of expression in these positive macrophages are greater for ESC_cyst_ (^∗^*P* < 0.05) (b). The percentage of macrophages expressing CD206 and the level of expression in these positive macrophages are not different between ESC_endo_ and ESC_cyst_ (b). Seven independent experiments (*n* = 4 biological replicates) were carried out in duplicates. Mean ± SD. Scale bars represent 50 *μ*m at 20x magnification.

To functionally characterize ESC_endo_ and ESC_cyst_, their effects on the differentiation of monocytes into macrophages were examined. Specifically, the morphology of the monocytes and their protein expression of CD14, CD163, and CD206 was examined following 7-day culture in the CM from ESC_endo_ and ESC_cyst_. Morphologically, ESC_cyst_ induced more spindle-shaped macrophages than ESC_endo_ (Figure 4(a)). Also, the percentage of macrophages expressing CD14 and CD163 and the MFI in these positive macrophages was significantly higher (*P* < 0.05) for ESC_cyst_ as compared to ESC_endo_ (Figure 4(b)). The percentage of macrophages expressing CD206 and the MFI in these positive macrophages was not significantly different (*P* > 0.05) between ESC_cyst_ and ESC_endo_ (Figure 4(b)).

Taken together, the results indicate that ESC_cyst_ may be functionally more immunosuppressive than ESC_endo_ based on their ability to induce more spindle-shaped M2 macrophages with a significantly higher (*P* < 0.05) level of expression of CD14 and CD163, both distinctive features of immunosuppressive M2 macrophages [20].

## 4. Discussion

Currently, it is unclear how endometriotic lesions avoid immunosurveillance in the pelvic cavity, and the cause of reduced immunosurveillance in the pelvic cavity is unknown. In addition, the phenotype and function of MSC in endometriotic lesions is not completely known. Herein, ESC from endometriotic ovarian cysts displayed all characteristics of MSC. We have shown that the ESC have a more immunosuppressive phenotype if located within endometriotic ovarian cysts compared to the eutopic endometrium and that they direct monocyte differentiation into immunosuppressive M2 macrophages. Taken together, ectopic MSC may contribute to reduced immunosurveillance in the pelvic cavity to allow immune escape of ectopic lesions and support their growth in endometriosis.

In agreement with previous findings, ESC_endo_ and ESC_cyst_ isolated from women with endometriosis met the criteria to be classified as MSC as they showed fibroblastic morphology, appropriate expression of surface markers, and differentiation into osteoblasts and adipocytes [27, 28]. In addition, and as shown before, ESC_endo_ formed CFU-F to a greater extent than ESC_cyst_ [29]. This could be because ESC_cyst_ grow in an ischemic microenvironment *in vivo* in the pelvic cavity, which may affect their proliferation and hence colony-forming ability.

Immunosuppressive MSC have been suggested to express high levels of immunosuppressive and low levels of immunostimulatory molecules, respectively. Therefore, we examined the expression of such molecules by ESC_endo_ and ESC_cyst_. Both protein and gene expression of IDO1, COX2, and HO1 was significantly higher in ESC_cyst_, suggesting that they may be more immunosuppressive phenotypically. Other studies have shown similar results, with higher gene expression of COX2 and HO-1 in ESC_cyst_ and stromal cells from peritoneal endometriotic tissue compared to ESC_endo_ [30, 31]. In contrast to these results, two studies reported that ESC_cyst_ expressed similar gene and protein levels of IDO1 as ESC_endo_ [32, 33]. It was also found that ectopic stromal cells may be more immunostimulatory than ESC_endo_ from healthy controls [34]. However, ESC_endo_ from healthy controls may introduce individual to individual variations in terms of the immunological microenvironment and endocrine factors [34]. Moreover, it is unclear if the ectopic stromal cells were from peritoneal endometriotic tissue or endometriotic ovarian cysts, which are two different types of endometriosis lesions [34]. Interestingly, in a differently designed study, IDO1 and COX2 gene expression was found to be higher in menstrual blood-derived stromal cells in women with endometriosis compared to healthy controls after culture in a transwell system with PBMCs [35]. In contrast to the latter study, herein, we used unstimulated ESC to reflect the *in vivo* environment more closely. We next examined if ESC_cyst_ were less immunostimulatory phenotypically than ESC_endo_ by studying their expression of CXCL12. Similar to a previous report [36], we found that protein expression of CXCL12 was significantly higher for ESC_cyst_ compared to ESC_endo_, but in contrast to herein, they stimulated their ESC with estrogen or progesterone [36]. However, the gene expression of CXCL12 was lower in ESC_cyst_. This discrepancy may be due to posttranscriptional and posttranslational processes, since weak correlations between mRNA and protein abundance have been described before [37]. Proteins, not genes, bestow cellular function, and therefore ESC_cyst_ seem to have a more immunostimulatory phenotype than ESC_endo_ [37]. CXCL12 is a ligand of the C-X-C chemokine receptor type (CXCR) 4, and through it, ESC_cyst_ may further increase levels of inflammation in the pelvic cavity by recruiting CXCR4-positive immune cells [38]. Interestingly, ESC_endo_ express CXCR4 and may hence be recruited to ectopic lesions by CXCL12, which may also possess nonimmune functions in endometriosis, such as promotion of tissue repair, angiogenesis, migration, invasion, and suppression of apoptosis [1, 38, 39], processes proposed to be involved in growth of ectopic lesions. ESC_cyst_ may be more immunostimulatory phenotypically than ESC_endo_ in response to fluctuations of levels of pathological inflammation in the pelvic cavity. In summary, these results imply that the inflamed pelvic cavity may induce ESC_cyst_ to become more immunosuppressive phenotypically to allow them to reduce inflammation and promote tissue homeostasis.

IDO1, COX2 via secretion of prostaglandin E2 (PGE2), and HO-1 have been suggested to be able to induce immunosuppressive M2 macrophages [13, 40–44]. Interestingly, endometriosis is a disease with profound macrophage involvement, with predominance of M2 macrophages in peritoneal endometriotic tissue, and endometriotic ovarian cysts that have been suggested to play a role in the pathology [14, 45–47]. Therefore, we examined the effects of ESC_endo_ and ESC_cyst_ on monocyte differentiation into macrophages. Morphologically, ESC_cyst_ were found to induce more spindle-shaped macrophages than ESC_endo_. Furthermore, ESC_cyst_ induced a significant increase in macrophages expressing scavenger receptors CD14 and CD163 compared to ESC_endo_. CD14 is involved in the uptake of apoptotic cells, CD163 is involved in the uptake of haptoglobin-hemoglobin complexes, and both have crucial roles in clearing up the pelvic cavity from apoptotic cells and heme-iron that accumulates by dying red blood cells, respectively [45, 48]. Elevated CD163 expression has been suggested to be a marker of M2c, a subtype of M2 macrophages involved in immunosuppression, matrix deposition, and tissue remodeling [20, 49–51]. Therefore, increased M2c levels may explain the extensive fibrosis that occurs in endometriotic lesions [52]. The macrophages expressed similar levels of the scavenger receptor CD206 after treatment with CM from ESC_endo_ and ESC_cyst_. CD206 is involved in inactivating inflammatory signals and may have a central role in the inflamed pelvic cavity [45]. In summary, this data shows that ESC_cyst_ may be more immunosuppressive functionally in comparison to ESC_endo_.

Two previous studies showed that ectopic stromal cells [53] or ESC_endo_ [47] polarized human macrophages or U937 monocytes stimulated with lipopolysaccharide into M2 macrophages, respectively. In the former study, macrophage expression of CD163 and CD209 and their intracellular expression and extracellular secretion of transforming growth factor-beta 1 (TGF-*β*1) and interleukin-10 (IL-10) was increased compared to macrophages treated with ESC_endo_ from healthy controls [53]. However, as discussed above, ESC_endo_ from healthy controls may not be an appropriate control as it may introduce individual to individual variations in terms of the immunological microenvironment and endocrine factors [53]. Moreover, it is unclear if the ectopic stromal cells were from peritoneal endometriotic tissue or endometriotic ovarian cysts, which are two different types of endometriosis lesions [53]. In the latter study, there was an increase in immunosuppressive cytokine IL-10 and a decrease in the expression of the costimulatory molecule CD86 by the M2 macrophages [47]. These studies suggested that IDO1 via IL-33 [53] secreted by ectopic stromal cells or ESC_endo_-derived fractalkine (FKN) [47], respectively, was driving the M2 macrophage polarization. Moreover, the M2-polarized macrophages significantly increased the viability and proliferation of ESC, decreased apoptosis of ESC [54], and enhanced the invasiveness of ESC_endo_ [47], suggesting that they may support the growth of ectopic lesions in endometriosis. In contrast to the aforementioned studies, we examined the effects of ESC_cyst_ on the ability of primary unstimulated human monocytes to differentiate into macrophages in comparison to ESC_endo_. To our knowledge, this is the first time that this has been performed.

**Figure 5:**
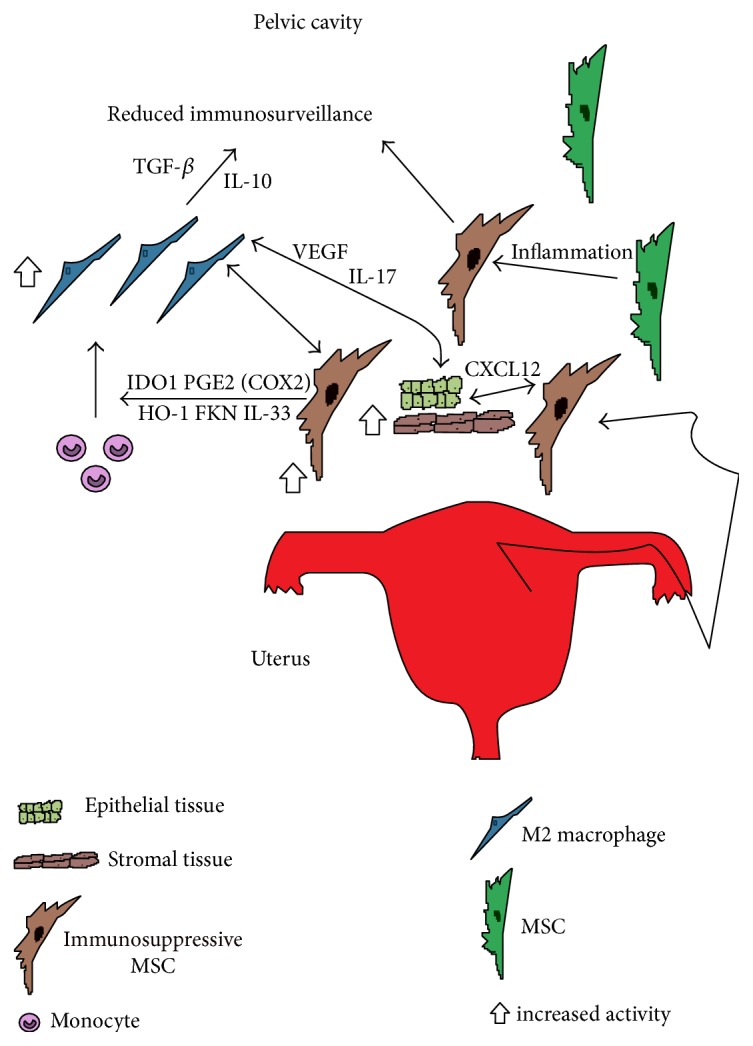
Schematic figure showing the role immunosuppressive ectopic MSC may be playing in the pathology of endometriosis. MSC along with stromal and epithelial endometrial tissue enter the pelvic cavity via retrograde menstruation. The highly inflammatory environment may induce MSC to become immunosuppressive to help promote tissue homeostasis. The recruited monocytes responding to the inflammatory environment and FKN, IL-33, IDO1, PGE2 via COX2, and HO-1 from immunosuppressive MSC may then polarize and differentiate into immunosuppressive M2 macrophages. Then, M2 macrophages may increase invasion of refluxed endometrial cells, repair ectopic lesions, and support their growth by inducing angiogenesis via their secretion of VEGF and IL-17 [58]. In addition, secretion of IL-10 and TGF-*β* by the M2 macrophages may suppress other recruited immune cells to reduce immunosurveillance in the pelvic cavity [58]. Also, ectopic MSC may directly support ectopic lesion growth by promotion of tissue repair, angiogenesis, migration, invasion, and suppression of apoptosis through CXCL12. The net effect may be large numbers of immunosuppressive MSC and M2 macrophages in the pelvic cavity that may directly support ectopic lesion growth, reduce immunosurveillance in the pelvic cavity, and reciprocally support each other's growth contributing to the development and progression of endometriosis. MSC: mesenchymal stromal cell; FKN: fractalkine; IDO1: indoleamine 2,3-dioxygenase 1; COX2: cyclooxygenase 2; HO-1: heme oxygenase 1; CXCL12: chemokine c-x-c motif chemokine ligand 12; VEGF: vascular endothelial growth factor; IL-17: interleukin 17; IL-10: interleukin 10; TGF-*β*: transforming growth factor-beta; IL-33: interleukin 33; PGE2: prostaglandin E2.

Interestingly, soluble factors in the CM from unstimulated ESC_cyst_ induced the M2 macrophage differentiation, indicating that stimulation of ESC_cyst_ by monocytes through paracrine mechanisms and direct contact are not required. IDO1 and HO1 can be secreted by MSC and, along with secreted PGE2 via COX2, may have been involved in ESC_cyst_ promoting M2 macrophage differentiation [55, 56]. M2 macrophages have been suggested to play a role in the pathology of endometriosis by recognizing initial ectopic lesions as wounds and initiating “healing” [45]. The wound healing properties of M2 macrophages may be important in skin wounds; however, they may be detrimental in endometriosis [57]. Moreover, M2 macrophage secretion of IL-10 and TGF-*β* may suppress other immune cells leading to reduced immunosurveillance in the pelvic cavity and hence protect ectopic lesions from immune clearance [58]. Therefore, it has been suggested that redirection of M2 macrophages to M1 macrophages may be a strategy to stimulate immune responses against ectopic lesions [45]. A schematic figure illustrating our proposed hypothesis for the role of ectopic MSC in the pathogenesis of endometriosis is shown in Figure 5.

The limited number of donors and presence of hormonal treatment did not affect the consistency of data between the four women with endometriosis being studied. Significant differences were observed, and meaningful conclusions could be made. A similar number of patients have been used in other studies [35, 59]. The stromal cells herein were unstimulated and unmodified but were cultured, which may alter their functional phenotype. Therefore, it would be interesting to study native stromal cells. Nevertheless, to our knowledge, this is the first *in vitro* study showing that ESC_cyst_ may have more immunosuppressive properties than ESC_endo_. This is an important finding that will improve our knowledge on the pathogenesis of endometriosis and may benefit development of new therapies.

## 5. Conclusion

In summary, immunosuppressive ectopic MSC may contribute to reduced immunosurveillance in the pelvic cavity. This may be in part by their immunosuppressive effects through M2 macrophages, which may subsequently support the growth of endometriotic ovarian cysts in endometriosis. This finding supports the retrograde menstruation and the stem cell theories by adding an immunosuppressive ectopic MSC component. Finally, we speculate that reducing the immunosuppressive effects of ectopic MSC to promote M1 macrophage and T Helper 1 responses may provide the necessary immunostimulation to remove ectopic lesions in the pelvic cavity and to potentially treat endometriosis.

## Figures and Tables

**Table 1:** Genes used in this study with the forward and reverse primers.

Gene	Forward primer	Reverse primer
*β*-Actin	AGCTACGAGCTGCCTGAC	AAGGTAGTTTCGTGGATGC
IDO1	GCATTTTTCAGTGTICTTCGCATA	TCATACACCAGCCGTCTGATAGC
COX2	ATCATAAGCGAGGGCCAGCT	AAGGCGCAGTTTACGGGTC
HO-1	CTTCTTCACCTTCCCCAACA	AGCTCCTGCAACTCCTCAAA
CXCL12	TTGACCCGAAGCTAAAGTGG	CCCTCTCACATCTTGAACCTCT
